# Improving automatic segmentation of liver tumor images using a deep learning model

**DOI:** 10.1016/j.heliyon.2024.e28538

**Published:** 2024-03-21

**Authors:** Zhendong Song, Huiming Wu, Wei Chen, Adam Slowik

**Affiliations:** a School of Mechanical and Electrical Engineering, Shenzhen Polytechnic University, Shenzhen, 518055, China; b Koszalin University of Technology, Koszalin, Poland

**Keywords:** Deep learning, Liver tumor, Loss function, Dice coefficient, Liver vessel segmentation, Image

## Abstract

Liver tumors are one of the most aggressive malignancies in the human body. Computer-aided technology and liver interventional surgery are effective in the prediction, identification and management of liver neoplasms. One of the important processes is to accurately grasp the morphological structure of the liver and liver blood vessels. However, accurate identification and segmentation of hepatic blood vessels in CT images poses a formidable challenge. Manually locating and segmenting liver vessels in CT images is time-consuming and impractical. There is an imperative clinical requirement for a precise and effective algorithm to segment liver vessels. In response to this demand, the current paper advocates a liver vessel segmentation approach that employs an enhanced 3D fully convolutional neural network V-Net. The network model improves the basic network structure according to the characteristics of liver vessels. First, a pyramidal convolution block is introduced between the encoder and decoder of the network to improve the network localization ability. Then, multi-resolution deep supervision is introduced in the network, resulting in more robust segmentation. Finally, by fusing feature maps of different resolutions, the overall segmentation result is predicted. Evaluation experiments on public datasets demonstrate that our improved scheme can increase the segmentation ability of existing network models for liver vessels. Compared with the existing work, the experimental outcomes demonstrate that the technique presented in this manuscript has attained superior performance on the Dice Coefficient index, which can promote the treatment of liver tumors.

## 1 Introduction

Liver tumor is one of the most serious tumors threatening human life, and its mortality rate ranks second in the world. Also, liver tumors are very common. How to effectively diagnose and treat liver tumors is one of the most concerned issues in the medical community all over the world. The automatic and precise segmentation of liver tumors based on computer vision and artificial intelligence technology has brought a solution to the above problems. Computed tomography (CT) is a crucial diagnostic instrument for detecting diseases, and its application extends to the detection and evaluation of liver malignancies. Precise segmentation of liver tissue from CT is an essential aspect of computer-assisted liver disease diagnosis and surgical preparation. Despite this, the automated detection and segmentation of liver tumors from CT images remains an enormous obstacle because of the indistinct boundaries and diminished contrast between tumor and healthy tissues in CT images.

Classification methods for medical images may be categorized into two distinct types: classification approaches founded on conventional machine learning and those based on deep learning methods. The first sorts usually use the gray value attributes of CT images and the statistical characteristics of lesion regions for classification. In contrast, classification techniques rooted in deep learning have the ability to learn features automatically from large amounts of data and use them for classification. Reference [1] employs three statistics-based techniques and two model-based methods for extracting texture features from images. These methods are applied individually as well as in pairs to classify the extracted texture features using Naive Bayesian. Reference [2] first utilizes the gray level co-occurrence matrix to extract image texture features and reduces the dimensionality, and then constructs a decision tree through the genetic algorithm to separate the fuzzy areas of the data and improve the recognition rate, and uses SVM for each node of the decision tree A classifier is trained and finally classified by the classifier.

Recently, deep learning has also shown its talents in image segmentation [[3], [4], [5]]. Incorporating this can substantially enhance the precision of tumor segmentation. Reference [6] used three deep convolutional networks (DNNs) to capture hepatic vascular features from different planes of CT image data, but the network could not segment data with large differences. However, it is difficult for traditional deep convolutional networks to accurately segment liver vessels. This is mainly due to the fact that the hepatic vessels tend to vary widely in shape and their terminal branches also exhibit irregular and minute features. Coupled with the limited open-source labeled data, the proportion of foreground and background pixels in the dataset is unbalanced, which makes the training easy to fall into a local optimum. Reference [7] proposed a symmetric fully convolutional network U-Net, which includes an encoder-decoder, and achieved good results in medical image segmentation. 3D-UNet [8] and V-Net [9] are dense fully convolutional networks designed for 3D biomedical image segmentation. Reference [10] improved the original 3DU-Net scheme, creatively added the residual module in the ResNet [11] network to the existing model, and constructed a new residual 3DU-Net for segmenting hepatic blood vessels from CT images.

Compared with 3D-Unet, V-Net adds a residual structure to the network and uses convolutional layers instead of pooling layers. The network can achieve end-to-end segmentation with few training samples and incomplete labels. The V-Net network structure can be divided into two parts, the decoder and the encoder. Through the skip connection structure, the features of the encoder are mapped to the decoder to make up for the lost spatial information during the upsampling process of the decoder. Although this structure retains the lost spatial features to a certain extent, due to the high similarity between liver blood vessels and surrounding tissues, it is still necessary to strengthen the localization ability of the network in order to accurately segment. V-Net implements a novel loss function, known as Dice Loss [9], which employs the dice coefficient to mitigate the influence of imbalanced foreground and background regions. Although the segmentation results have improved, it is still unable to accurately segment small targets. In order to improve the performance of the existing model, this paper optimizes based on V-Net, and designs a new network model to realize the automatic segmentation of liver vessels. Specifically, the improvements are as follows: (1) Change the network structure and introduce pyramidal volumes in the skip connection process Blocks, which are used to fuse local and global image content to reduce information loss. (2) Introduce multi-resolution deep supervision in the network, and divide the liver blood vessels into multi-resolution feature maps, which is regarded as multi-task learning. The parameters of different supervision paths are updated independently without interfering with each other, making the training of each path more effective. Then the feature maps of different resolutions are combined to output the final prediction result. (3) To solve the challenge that the foreground and background voxels are highly unbalanced, the Tversky loss function [12] is used.

## 2 Background

### 2.1 Liver imaging and vessel segmentation

Medical imaging plays a crucial role in the diagnosis and treatment of liver diseases. Among various imaging modalities, Computed Tomography (CT) and Magnetic Resonance Imaging (MRI) stand out for their ability to provide detailed visualization of liver anatomy. CT scans are particularly valued for their high spatial resolution, offering clear cross-sectional images of the liver, including vascular and parenchymal structures. MRI, known for its superior contrast resolution, excels in differentiating soft tissues and is less reliant on contrast agents than CT. These imaging techniques, however, face several challenges.

One of the primary challenges in liver imaging is the variability in imaging parameters and patient-specific factors, such as movement or body habitus, which can significantly affect image quality. Inconsistencies in contrast agent distribution further complicate the interpretation of these images. These factors underscore the need for sophisticated image processing techniques for accurate liver anatomy representation. Liver vessel segmentation, a critical aspect of liver imaging, is particularly challenging due to the liver's complex vascular architecture and the presence of various pathologies. The heterogeneity in vessel size, branching patterns, and course, along with pathological changes like tumors or cirrhosis, adds to the complexity of segmentation. The low contrast between hepatic vessels and surrounding parenchyma often results in poor boundary delineation. Additionally, the partial volume effect, where a single voxel may contain multiple tissue types, leads to segmentation inaccuracies. Addressing these challenges requires the development of robust, adaptive, and highly accurate segmentation algorithms capable of handling the intricate details of liver vasculature.

### 2.2 Deep learning in medical imaging

Deep learning, a branch of artificial intelligence and machine learning, has shown remarkable success in medical imaging. It involves training deep neural networks on large datasets, enabling them to learn features and patterns relevant to specific tasks. In medical imaging, deep learning techniques, particularly Convolutional Neural Networks (CNNs), have been extensively used for tasks like segmentation, classification, and anomaly detection. These methods have demonstrated superior performance over traditional image processing techniques, especially in handling complex and variable patterns inherent in medical images. Common deep learning algorithms used in medical imaging include U-Net, V-Net, and their variants, which have been specifically designed for volumetric (3D) data segmentation. These algorithms have shown significant improvements in segmentation accuracy, efficiency, and robustness, making them particularly suitable for complex tasks like liver vessel segmentation. Compared to traditional methods, deep learning algorithms can automatically learn and adapt to the variability in liver vessel appearance, size, and shape, leading to more accurate and reliable segmentation results.

CNNs are a class of deep neural networks, most commonly applied to analyzing visual imagery. They are characterized by their convolutional layers, which apply a series of learnable filters to the input image, capturing spatial hierarchies and patterns. These layers are typically followed by pooling layers, which reduce the spatial dimensions of the data, and fully connected layers, which make decisions based on the features extracted by the convolutional layers. CNNs are particularly effective in image segmentation tasks due to their ability to learn location-invariant features and their efficiency in handling large images.

V-Net, an extension of the CNN architecture, is specifically designed for volumetric image segmentation, making it highly relevant for tasks like liver vessel segmentation in CT or MRI data. It extends the concept of CNNs to 3D, allowing for the analysis of volumetric data directly. V-Net improves upon traditional CNNs by incorporating residual learning and a novel objective function, which helps in dealing with the class imbalance problem common in medical image segmentation. The architecture of V-Net is similar to that of U-Net, another popular segmentation network, but it is specifically optimized for 3D data with a focus on capturing the complex spatial relationships present in volumetric images.

## 3 Related work

Over the past few decades, the methods for liver segmentation in CT images were mainly designed based on the intensity, texture, and other information of abdominal CT images, including region growing, thresholding, and level set algorithms. Since the traditional region growing algorithm relies on artificially selected seed points, based on this, Thomaz et al. optimized the traditional region growing algorithm [13], using the t position scale distribution to obtain the position and scale parameters of the target area for segmentation. This method does not rely on artificially selected seed points, but it does not have wide adaptability. Based on this method, Tan et al. suggested an adaptive algorithm for region growth with a curvature strategy, which has become one of the most promising tools for tumor segmentation [14]. However, the efficiency of this method depends on the homogeneity of the tissue, and under-segmentation sometimes occurs. Arica et al. [15] investigated the use of the region growing technique to differentiate similar and dissimilar liver tissues in CT images. This involved selecting a seed point for every connected region, and use the region growing algorithm to segment. The above problems are effectively solved. In reference [16], Zeng et al. used double Gaussian filtering and K-means clustering hybrid algorithm to apply CT liver vessel segmentation. With two different segmentation strategies, the method achieves an average accuracy of 98.2%, a Dice of 73.0% and an RMSD of 2.56 mm, but is not universally applicable.

In order to improve the applicability, Chartrand et al. initially set 3–10 seed points, and manually adjusted the parameters to improve the accuracy. In this method, each image processing takes about 3min [17]. Ever since its inception in 1988 by Osher and Sethian, the level set algorithm [18] has found extensive applications in image segmentation, modeling, and computer vision. Several researchers have conducted studies centered on the level set methodology. For instance, Zheng et al. [19] introduced a consolidated level set method (LSM) for the segmentation of liver tumors based on liver CT image area and edge information. The algorithm was verified on the 3Dircadb dataset, and the results showed that the VOE (voxel overlap error) was 11.9%. The average surface distance (ASD) is 1.3 mm, the volume difference (RVD) is 18.5%, and the maximum surface distance (MSD) is 4.6 mm, which improves the accuracy of liver tumor segmentation. In addition, Li et al. [20] used the level set algorithm combining likelihood energy and edge energy. The algorithm was verified on 18 clinical data sets and showed that RVD was (−8.1 ± 2.1) % and ASD was (2.4 ± 0.8) mm with an MSD of (7.2 ± 3.1) mm, which results in an improvement in segmentation performance.

In order to overcome the intensity inhomogeneity in liver CT images, Li et al. [21] first used sparse shape components to roughly segment the liver, then used level set to optimize the segmentation results, and finally verified the segmentation performance on the dataset 3Dircadb, the average of the method This method selects four indicators such as ASD, MSD, VOE and RVD for performance evaluation. Among them, the average surface distance is 1.6 mm. The maximum surface distance is 27.2 mm. The VOE and RVD are 9.2% and 0.5% respectively. and have more accurate segmentation effects on normal liver and pathological liver. Reference [22] builds a classifier trained by a convolutional neural network (CNN) through the determined region of interest, and then subdivides the tumor in the classifier, but this method relies on the artificially calibrated region in advance. To this end, Wang et al. [23] proposed a new algorithm to segment tumors. This method extracts the texture features of different regions of the image through support vector machines and improved level sets, and uses SVM classifiers to detect suspicious lesion regions. Thus, the complete CT images can be acurired for tumor segmentation.

Traditional machine learning methods generally have the following problems: 1) It takes a long time to extract features such as texture and shape of the image, and the selection of effective features needs to be carefully considered; 2) The calculation time spent by different dimensionality reduction methods and the obtained results Inconsistent, improper dimensionality reduction methods may cause data redundancy; 3) There is a lack of research on the depth characteristics of lesion regions, and because the lesion regions are different, it is difficult to classify too small lesion regions by traditional features.

The method based on deep learning solves the problem that traditional machine learning methods need to spend time manually extracting image features and selecting an appropriate method for dimensionality reduction, and at the same time can obtain advanced features. Deep learning technology has demonstrated remarkable success in the realm of image analysis, particularly in medical imaging. More researchers have turned their research ideas to deep learning methods. Reference [24] proposes a synergistic deep learning model (SDL). Meanwhile, to further improve the model performance, the authors combined several deep convolutional neural networks (DCNN) [25] with SDL. Specifically, after using DCNNs to extract image features, the outputs of multiple DCNNs are combined and input into SDL together. In Ref. [26], an end-to-end discriminative network framework for classifying liver lesions was introduced. This involved extracting features from convolutions of various sizes using Inception V3 and leveraging transfer weights pre-trained on ImageNet. The model's performance was further enhanced by pooling operations executed via parallel processing. On the other hand, reference [27] incorporated extreme learning machine (ELM) into the segmentation of liver images. First, the authors employ convolutional neural networks to automatically extract features from images to obtain more abstract and high-level features. The extreme learning machine can rapidly learn the inherent characteristics of the data, so as to achieve better generalization performance. In addition, ELM can effectively avoid the overfitting problem. The authors used extreme learning machines for image classification to segment the liver.

Liang Liming et al. [28] enhanced the segmentation of retinal blood vessels by employing a densely deformable structure that effectively captures multi-scale information and structural shapes. In 2019, Li et al. [29] introduced the Context-Aware Sensitive U-Net (CASU), which incorporates an integrated attention gate (AGs) before concatenating corresponding features in the encoder and decoder of U-Net. Focusing on channel relationships, Hu et al. [30] proposed a novel architectural unit named the Squeeze-and-Excitation (SE) block. This module adaptively recalibrates channel-wise feature responses by explicitly modeling interdependencies between channels. However, Pereira et al. [31] observed that the SE block, recalibrating information only in channels, was not ideally suited for image segmentation. Therefore, they proposed a recalibration block that combines feature reorganization with spatial adaptability, offering a more suitable approach for semantic segmentation. Reference [32] introduces a novel network structure combining UNET and LSTM models with a mixed loss function, addressing morphological variety and category imbalance in brain tumor segmentation. Utilizing multimodal brain tumor images, the proposed method demonstrates superior accuracy in segmenting various tumor lesions, as evidenced by high DSCs, sensitivities, and specificities. Although deep learning has been applied to numerous medical imaging tasks, the unique properties of the vascular system may render existing structures suboptimal and not immediately deployable for these specific tasks [33]. Consequently, there is a need to explore alternative network architectures and training strategies tailored to these unique requirements.

## 4 Our proposed network model

### 4.1 Improvement of V-Net network framework

The original V-Net is a dense symmetric network with five resolution layers. The encoder on the left is used to extract features from the image, and the decoder on the right combines the information of the encoding path with the upsampled multi-scale The feature map information is combined, and finally restored to the original resolution, and pixel-by-pixel classification is performed on this basis. In order to prevent overfitting in the training process and reduce the amount of calculation, the original network's 5 × 5 × 5 convolution kernel is substituted with a 3 × 3 × 3 convolution kernel, and the PReLU activation function is utilized and adopted to the entire network. To avoid network overfitting, the dropout layer is introduced in the network model.

**Fig. 1:**
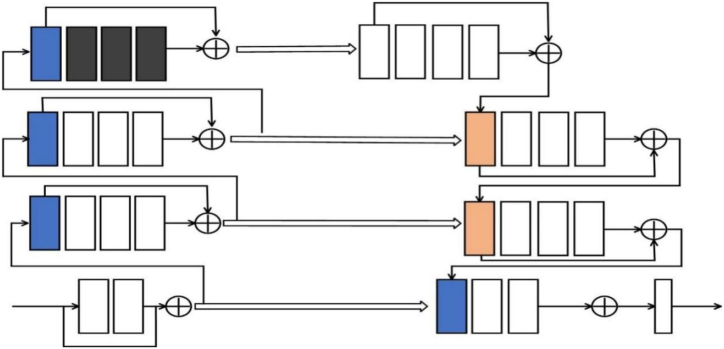
Structure of our proposed network model.

The improved network structure is presented in Fig. 1. The encoder located on the left and the decoder positioned on the right each contain a four-layer network structure. The original V-Net network uses 2 × 2 × 2 with a step size of 2 multiple times in the encoder. The convolution kernel downsamples the feature map. While down-sampling can augment the receptive field, it may reduce the spatial resolution, resulting in the loss of feature map details. Therefore, this paper deletes the last layer of the original V-Net encoder, and introduces a dilated convolution in the fourth layer of the encoder to compensate for the decrease in the receptive field of the feature map caused by the reduction of the downsampling layer. The expanded convolution kernel is calculated in Equation (1):

(1) $$\begin{array}{c} k_{d}=k+\left(k-1\right)\left(r-1\right) \end{array}$$

which we can see: k refers to the dimension of the initial convolution kernel, whereas r denotes the expansion rate, and the expansion rates in the three-dilated convolutions are 3, 4, and 5, respectively. The dilated convolution operation can increase the receptive field without using downsampling to shrink the feature map, and by adjusting the expansion rate value in the dilated convolution, feature information of different scales in the feature map can be extracted. The feature channels can be doubled at each successive layer along the encoding path for more accurate and adequate learning of deep features. The decoding pathway employs a 2 × 2 × 2 deconvolution with a stride of 2 to upsample each layer, and halving the number of feature channels. Finally, the output layer uses a 1 × 1 × 1 convolution to fine-tune the number of channels in the feature map.

### 4.2 Pyramid convolution block

**Fig. 2:**
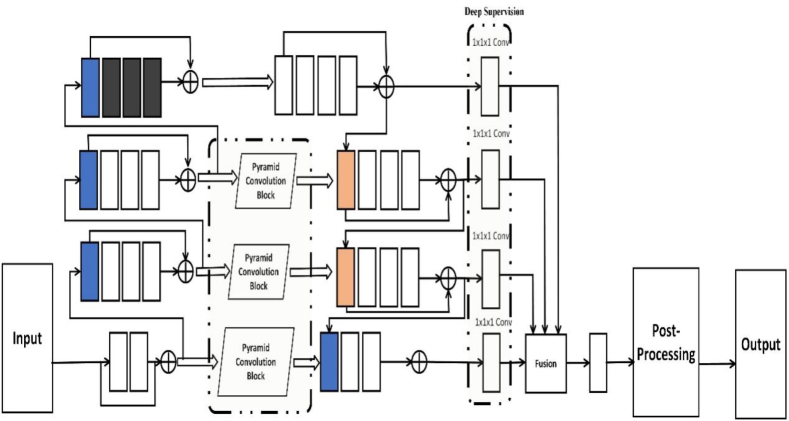
Overall structure of our design.

Semantic segmentation is a classification at the pixel level, which requires pixel-by-pixel positioning and classification. The blood vessels in the liver are intricate and have low contrast with the surrounding tissue, so it is necessary to enhance the classification and localization capabilities of the network. Reference [34] pointed out that in addition to the inherent spatial positioning ability of large kernel convolution, it can also improve the voxel classification ability. To prevent overfitting of the network due to excessive network parameters caused by the use of large convolution kernels in the main body of the network, this paper introduces pyramidal convolution blocks in the horizontal connection process of each layer. The connection locations of the pyramid convolution blocks are shown in Fig. 2, represented by parallelogram boxes. The structure of the pyramidal convolutional block is constructed in parallel with 3 × 3 × 3, 5 × 5 × 5 and 7 × 7 × 7 convolutional blocks. Besides, the residual structure is added, and a 1 × 1 × 1 convolution is attached after the residual structure. The formula of the residual structure is shown in Equation (2):

(2) $$\begin{array}{c} x_{L+1}=x_{l}+\sum_{i=l}^{L-1}F\left(x_{i},W_{i}\right) \end{array}$$

in the formula: $x_{l}$ is the input feature; $\sum_{i=l}^{L-1}F\left(x_{i},W_{i}\right)$ is the accumulated residual function, which represents the output feature of each layer after 1 × 1 × 1 convolution and pyramidal convolution block; $W_{i}$ refers to a collection of weights that correspond to the residual unit.

The features output from the encoder are propagated to the decoder through the pyramidal convolution block, which helps the network capture the spatial feature information of liver vessels from different scales to generate more discriminative features. Convolutions of different scales can effectively fuse local and global image content, and reduce information loss, thereby enhancing the localization ability of the network.

### 4.3 Multi-resolution depth supervision

In this study, we have refined the deep supervision mechanism, as initially described in Reference [35], to suit the specific requirements of liver vessel segmentation. This technique is designed to address several common challenges in neural network training, such as the prevention of gradient vanishing or exploding, enhancing the speed of network convergence, reducing the likelihood of overfitting, and improving the model's generalization capabilities.

Our model employs a multi-task learning framework, where the segmentation of liver vessels is conceptualized as a multi-layered process. This involves the generation of feature maps at various resolutions across different hidden layers of the network, ranging from lower to higher levels of abstraction. By integrating multi-scale feature map fusion, we aim to capture more detailed and informative representations from these layers, thereby increasing the segmentation accuracy. Distinct from traditional deep supervision methods, our approach supervises feature maps at multiple resolutions through several pathways within a four-layer network structure. The process is as follows:

**Dimensionality Reduction:** In the decoder section of the network, each layer's feature map undergoes a dimension reduction process using a 1 × 1 × 1 convolution, except for the lowest layer.

**Feature Map Fusion:** For all layers above the first, the dimension-reduced feature maps are upsampled using trilinear interpolation and then fused with the dimension-reduced feature maps from the preceding layer. This fusion process is repeated in a stepwise manner, leading to the formation of the final feature map.

**Softmax and Loss Calculation:** The dimension-reduced feature maps from all but the lowest layer are processed through a Softmax layer. The loss for each map is then calculated against labels of matching resolution, derived through trilinear interpolation downsampling. This setup allows for independent loss calculation and weight updating across multiple network pathways. The total loss function is expressed in Equation (3):

(3) $$\begin{array}{c} L_{total}=L_{all}+\sum_{d=1}^{3}L_{d} \end{array}$$

In the formula: $L_{all}$ is the loss function after fusion; $L_{d}$ is for the dth layer. Because multi-resolution deep supervision makes liver vessel segmentation a multi-task learning process, and the parameter update of each path is equally important, so the loss weights of different supervised paths should be equal.

**Output Prediction:** The final segmentation prediction is generated after passing the last feature map through the Softmax layer.

This multi-resolution depth supervision approach allows for a more nuanced and effective computation of the total loss function, taking into account the diverse resolutions and hierarchical nature of the feature maps. Since each path has its own resolution training target, the parameters of different supervised paths are initialized randomly. In this way, the parameters are updated independently without interfering with each other, so as to prevent the network from falling into the same local optimum, so that the training of each path contributes to a better semantic expression of the network. At the same time, the network can learn multi-level context information for the fusion of feature maps of different resolutions, which helps to subdivide the prediction results, so that the network has the potential to segment small liver vessels.

### 4.4 Loss function

To deal with the category imbalance, this paper chooses the Tversky loss function. Tversky defines it as shown in Equation (4):

(4) $$\begin{array}{c} L_{T}\left(P,G,\alpha ,\beta \right)=\frac{\left|P\cap G\right|}{\left|P\cap G\right|+\alpha \left|P-G\right|+\beta \left|G-P\right|} \end{array}$$

in the formula: P is the prediction label; G is the annotation label; |P∩G| is the total number of foreground voxels that are truly classified; |P−G| represents the number of false positives that are over-segmented voxels; |G−P| is the total number of false negatives that are under-segmented voxels; |P−G|+|G−P| means the total number of all wrongly classified voxels. Among them, the trade-off between false positives and false negatives can be controlled by adjusting α and β. The definition of the Tversky loss function is Equation (5):

(5) $$\begin{array}{c} T\left(\alpha ,\beta \right)=\frac{\sum_{i=1}^{N}p_{0i}g_{0i}}{\sum_{i=1}^{N}p_{0i}g_{0i}+\alpha \sum_{i=1}^{N}p_{0i}g_{1i}+\beta \sum_{i=1}^{N}p_{1i}g_{0i}} \end{array}$$

in the formula: in the output of the Softmax layer, $p_{0i}$ represents the probability that voxel i belongs to the foreground (hepatic blood vessel); $p_{1i}$ represents the probability that voxel i belongs to the background (non-liver blood vessel); $g_{0i}$ and $g_{1i}$ represent the foreground and background volume in the label The label of prime i, whose values are 1 and 0, respectively. The loss gradient with respect to $p_{0i}$ and $p_{1i}$ in above equation can be calculated in Equations (6), (7), respectively:

(6) $$\begin{array}{c} \frac{\text{dT}}{dp_{0i}}=2\frac{g_{0j}\left(\sum_{i=1}^{N}p_{0i}g_{0i}+\alpha \sum_{i=1}^{N}p_{0i}g_{1i}+\beta \sum_{i=1}^{N}p_{1i}g_{0i}\right)}{{\left(\sum_{i=1}^{N}p_{0i}g_{0i}+\alpha \sum_{i=1}^{N}p_{0i}g_{1i}+\beta \sum_{i=1}^{N}p_{1i}g_{0i}\right)}^{2}}-\frac{\left(g_{0j}+\alpha g_{1j}\right)\sum_{i=1}^{N}p_{0i}g_{0i}}{{\left(\sum_{i=1}^{N}p_{0i}g_{0i}+\alpha \sum_{i=1}^{N}p_{0i}g_{1i}+\beta \sum_{i=1}^{N}p_{1i}g_{0i}\right)}^{2}} \end{array}$$

(7) $$\begin{array}{c} \frac{\text{dT}}{dp_{1i}}=\frac{-\beta g_{1j}\sum_{i=1}^{N}p_{0i}g_{0i}}{{\left(\sum_{i=1}^{N}p_{0i}g_{0i}+\alpha \sum_{i=1}^{N}p_{0i}g_{1i}+\beta \sum_{i=1}^{N}p_{1i}g_{0i}\right)}^{2}} \end{array}$$

The Tversky loss function does not need to balance the weights for training. By tuning the hyperparameters α and β, the trade-off between false positives and false negatives can be controlled. It is verified by experiments that when α = 0.3 and β = 0.7, the effect is the best, and the segmentation accuracy is the highest.

### 4.5 The process of post-processing

The post-processing step is to further process the liver vessels predicted by the network model to enhance the model's performance. In this step, the volume of each connected domain needs to be calculated first. To distinguish between noise and actual liver vessels, we set an empirical threshold: connected domains smaller than 180 mm³ are considered as noise. This threshold is a balanced decision based on our experience, as a larger threshold might exclude significant disconnected vessels, while a smaller one could retain excessive noise. The disconnected blood vessels in our context refer to interruptions in vascular continuity observed in imaging, which may arise from limitations in imaging resolution or contrast, algorithmic imperfections in image processing and segmentation, or physical and pathological changes in the vessels. Adding a post-processing step can reduce incorrect noise removal to reduce misclassification in segmentation.

## 5 Experimental results

### 5.1 Experimental settings

In our study, we have utilized two publicly available datasets to conduct our experiments, specifically focusing on the domain of liver vessel segmentation. The first dataset employed is the LiTS17 (Liver Tumor Segmentation) dataset, a well-known resource in the medical imaging community. This dataset comprises 201 abdominal CT scans, each annotated with precise liver and liver tumor labels. For the purposes of our research, we have extracted the liver labels from this dataset to train and refine our liver vessel segmentation model. The second dataset we have utilized is the Hepatic Vessel dataset from the 2018 MICCAI challenge [36], a part of the Medical Segmentation Decathlon (MSD) competition held during the Medical Image Computing and Computer Aided Interventions (MICCAI) Conference in 2018. Task08_HepaticVessel, a specific subset of this challenge, provides a comprehensive collection of datasets that are widely used as a benchmark for evaluating various vessel segmentation methods. This task is instrumental in fostering research and development in the field of liver vessel segmentation, offering a unified and standardized dataset for researchers worldwide. The Hepatic Vessel dataset includes 303 abdominal CT scans, complete with accurate annotations of liver vessels and gallbladder structures. In our research, we have meticulously extracted the blood vessel labels from this dataset for a thorough experimental analysis. The utilization of these datasets not only provides a robust foundation for our experimental procedures but also ensures that our research is aligned with current standards and practices in the field of medical image segmentation. The experimental hardware environment used in this article is lntel Xeon E5-1603 CPU, 16 GB of memory, and the GPU of model GTX1080ti to accelerate the image processing.

In order to evaluate the performance of the method in this paper, we employed a k-fold cross-validation approach. The datasets were divided into k subsets, and in each round of validation, one subset was used as the test set while the others were used for training. This process was repeated k times, with each subset being used exactly once as the test set. The following evaluation indicators are used in this paper: the coincidence rate (Dice) of the overall segmentation accuracy evaluation, voxel segmentation accuracy, sensitivity, and specificity. The Dice coefficient (Dice) is a measure of the overlap of two images, indicating the coincidence rate of the blood vessel drawn by the radiologist and the blood vessel segmented in this paper, and the value range is between [0, 1]. The goal of Dice is to maximize the overlap between the radiologist's delineation of liver vessels and the segmented vessels, and the larger the value, the more accurate the segmentation.

The definitions of Dice, Accuracy, Sensitivity, and Specificity are given in Equations (8), (9), (10), (11) respectively:

(8) $$\begin{array}{c} Dice=\frac{2TP}{2TP+FP+FN} \end{array}$$

(9) $$\begin{array}{c} Accuracy=\frac{TP+TN}{TP+TN+FP+FN} \end{array}$$

(10) $$\begin{array}{c} Sensitivity=\frac{TP}{TP+FN} \end{array}$$

(11) $$\begin{array}{c} Specificity=\frac{TN}{TN+FP} \end{array}$$

Among them, TP means that how many voxels are segmented into blood vessels correctly (true positive); TN means that how many voxels are segmented into the background correctly (true negative); while FP means how many voxels are segmented into blood vessels incorrectly (false positive); FN means how many voxels in total are segmented as background incorrectly (false negatives).

In our experimental setup, we chose to implement a 10-fold cross-validation on both the LiTS17 and MICCAI Hepatic Vessel datasets. Before the training starts, the data preprocessing is required. The process of preprocessing includes the following three steps: (1) Limit the CT value of the picture to the range of 0HU∼400HU, focusing on the intensity range of the liver; (2) Normalize the image to zero mean and unit variance, and use coordinate transformation and cubic sample Strip interpolation converts the data into 1 mm thick slices; (3) The improved model proposed in this paper is trained using the LiTS17 dataset. After the model converges, use the output network model to apply it to the MICCAI Hepatic Vessel dataset, and make a liver mask. The size of the liver mask is obtained by expanding the segmented liver label by 20 mm outward in each dimension, and the same liver mask area is cut out on the CT image and the real label.

For training network parameter settings, this paper chooses a typical Adam optimizer in V-Net network training. Due to the small number of training samples, in order to prevent overfitting and improve the generalization ability of the model, in addition to data enhancement, this paper also adds a dropout layer at the end of the residual structure of each layer in the network, and the parameter is set to 0.5. The initial learning rate is 0.0001. Considering the computing resources, the batch size of the input network is set to 1.

### 5.2 The effect of different loss functions on the performance of our network model

This paper uses the Tversky loss function to improve the performance of existing models to deal with class imbalance. In order to evaluate the effectiveness of the loss function selected in this paper, the model in this paper is combined with different loss functions and the experimental results are compared. This paper selects 4 loss functions to compare with the loss function used in this paper. The four loss functions are: Dice loss function (benchmark method); Generalized Dice Loss; Binary Cross Entropy Loss (BCE Loss for short) and Focal Loss [37]. The Tversky Loss and Generalized Dice Loss selected in this paper are all passed in Dice Loss Weights are added to the function to enable the network to pay more attention to the region to be segmented with a small proportion of voxels when the gradient is updated. α and β are hyperparameters of Tversky Loss, and their values need to be adjusted manually during model learning. The weight item added by Generalized Dice Loss is controlled by the proportion of the area to be segmented. The smaller the voxel proportion, the greater the weight. BCE Loss is one of the most commonly used loss functions for binary segmentation tasks. It evaluates model performance based on the cross-entropy between predicted and ground truth segmentation masks. In the field of deep learning, binary cross-entropy loss is widely used in binary classification tasks, such as image segmentation, object detection, etc. FocalLoss is a loss function for binary classification tasks that handles imbalanced datasets. It makes the model pay more attention to samples that are difficult to classify by weighting the prediction scores. It is mainly used to deal with the category imbalance problem in the target detection task. By reducing the weight of easy-to-classify samples, it makes the weight of difficult-to-classify samples larger, thereby improving the classification accuracy of difficult-to-classify samples.

**Fig. 3:**
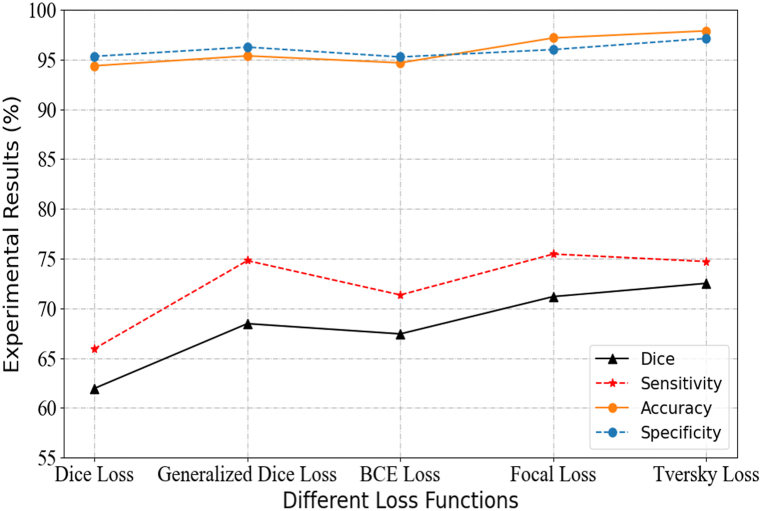
Evaluation on effect of different loss functions on the experimental results.

The experimental results are shown in Fig. 3. As can be seen from the figure, for Sensitive, the experimental results of the two loss functions of Generalized Dice Loss and Focal Loss are higher than Tversky Loss. Specifically, Generalized Dice Loss is only 0.09% higher than Tversky Loss in Sensitive. Focal Loss is 0.75% higher than Tversky Loss on Sensitive. However, for the other three metrics, Tversky Loss is higher than the other four loss functions. Taking Dice Coefficient as an example, Tversky Loss is 1.34% higher than Focal Loss and 4.06% higher than Generalized Dice Loss. To assess the stability of our model, we focus on the Dice Similarity Coefficient as a key indicator. This paper presents an analysis of the variability in experimental results across five distinct loss functions under the context of 10-fold cross-validation evaluations. The corresponding DSC values (i.e., DSC ± STD) are 61.96 (±6.4)%, 68.47 (±5.7)%, 67.44 (±8.5)%, 71.19 (±7.3)%, and 72.53 (±5.9)% respectively, for these five functions Dice Loss, Generalized Dice Loss, Binary Cross Entropy Loss, Focal Loss, and Tversky Loss. The observed variation in these values suggests that different loss functions exhibit varying degrees of sensitivity to model errors. Therefore, when the model structure is optimally selected, Tversky Loss performs better than the other four loss functions in terms of overall index evaluation, and has better comprehensive performance.

### 5.3 Comparative experiments of our method with different methods

The proposed approach is compared with existing research to evaluate the performance. The proposed approach is compared with other 11 methods. The Dice Coefficient is adopted as the indicator for performance comparison. The 11 methods involved in the comparison are: 3D-UNet [8], V-Net [9], LSTM-Unet [38], Multi Planar-UNet (MP-UNet for short) [39], Spider-UNet [40], UMCT [41], nnU-Net [42], 3DRes-Unet [10], FCN-RNN, 3D-VGN [43] and 3D Graph-Connectivity Constrained Network (3D-GCCN for short) [44]. The summary of the key features for all the compared methods are listed in Table 1.

**Table 1:** Summary of the all compared methods.

Method Name	Key Features
3D-UNet [8]	Dense fully convolutional network for 3D biomedical image segmentation
V-Net [9]	Adds a residual structure and uses convolutional layers instead of pooling
LSTM-Unet [38]	Integrating bidirectional LSTM with UNet. Designed for retinal vessel segmentation, applied to liver vessels in this study
MP-UNet [39]	Utilizes multi-planar training for efficient 3D representation in 2D U-Net architecture
Spider-UNet [40]	U-Net-based, integrates LSTM for 3D blood vessel segmentation with enhanced inter-slice connectivity
UMCT [41]	Utilizes multi-view co-training and uncertainty-aware label fusion for effective semi-supervised learning in 3D medical imaging
nnU-Net [42]	Automated method with hyperparameter search and data augmentation, end-to-end joint loss function
3DRes-Unet [10]	Integrates ResNet's residual blocks into 3DU-Net
FCN-RNN	Integrates Fully Convolutional Networks with Recurrent Neural Networks for enhanced medical image segmentation
3D-VGN [43]	Combines graph neural network and CNN for vessel segmentation
3D-GCCN [44]	Integrates graph neural networks into CNNs, models vessel connectivity with GAT

3DU-Net and V-Net are two dense fully convolutional networks specifically designed for 3D biomedical image segmentation. Compared with 3D-Unet, V-Net adds a residual structure to the model and exploits a convolutional layer instead of a pooling layer. 3DRes-Unet applies the residual structural block from ResNet network into 3DU-Net, aiming to segment liver vessels from CT images. The LSTM-Unet model is not designed for the segmentation of liver vessels, but for segmenting retinal vessels. Retinal vascular density can reflect the characteristics of ocular diseases. Doctors can diagnose eye diseases, including glaucoma, by looking at the characteristics of blood vessels in the retina. The experimental evaluation in this paper applies the LSTM-Unet model for liver vessel segmentation using the dataset.

nnUNet is an automated method for medical image segmentation, which is applicable to multiple medical image datasets and tasks. The method uses automated hyperparameter search and data augmentation techniques, and adopts an end-to-end training method based on a joint loss function, which can increase the segmentation accuracy largely. 3D-VGN is a 3D extension method based on VGN, which aims to solve the problem of vessel segmentation in 3D medical images. This method uses the embedding mode of graph neural network (GNN) and CNN. By inserting the graph convolution layer in GNN between the encoder and decoder of CNN, it achieves the goal of blood vessel segmentation. Information passing and feature extraction. Good segmentation results have been achieved by this method on several publicly available 3D blood vessel segmentation datasets.

3D-GCCN integrates graph neural networks into general convolutional neural networks by exploiting prior connectivity. The graph connection information of blood vessels is modeled by GAT. When integrated with 3D-UNet, hepatic vessel segmentation is effectively improved during training without increasing hardware and time costs during inference.

**Fig. 4:**
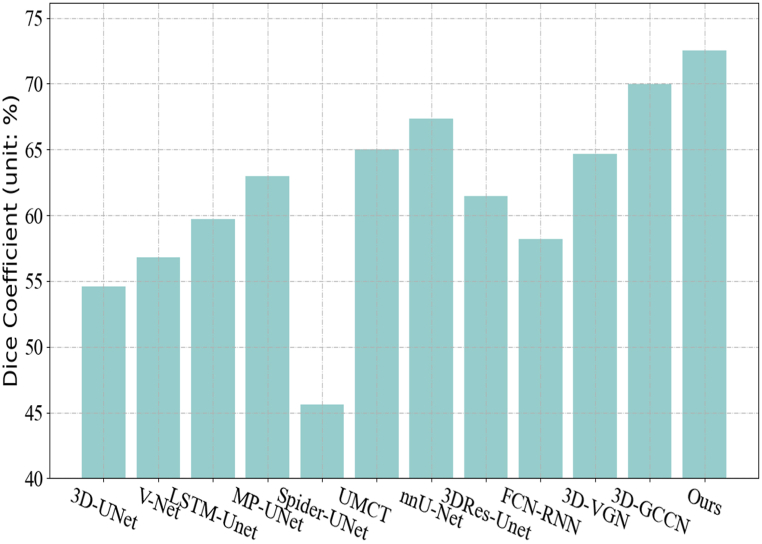
Experimental results comparison of our proposed method with different methods.

The experimental results are displayed in the form of histograms, as shown in Fig. 4. Among them, each model is evaluated according to the average cross-entropy (Dice Coefficient). A higher index value indicates a better segmentation result. The horizontal axis is all methods involved in the comparison. Experimental results show that our method performs best with an average cross-entropy index of 72.53. Followed by methods such as UMCT, nnU-Net and Multi Planar-UNet. While Spider-UNet has the worst performance. In addition, the performance of 3D-UNet and V-Net methods is relatively close. From these results, it can be concluded that methods using 3D deep convolutional neural networks combined with advanced techniques such as GNN or RNN are generally better suited to the needs of medical image segmentation tasks than using traditional models such as U-Net or FCN. Besides, Multi-Planar UNet is able to exploit multi-planar information to improve segmentation performance. Although both UMCT and nnU-Net achieve high segmentation performance, the complexity of these methods may lead to higher computational cost and longer training time. Therefore, the balance between performance and computational cost should be taken into consideration when choosing a medical image segmentation model.

**Fig. 5:**
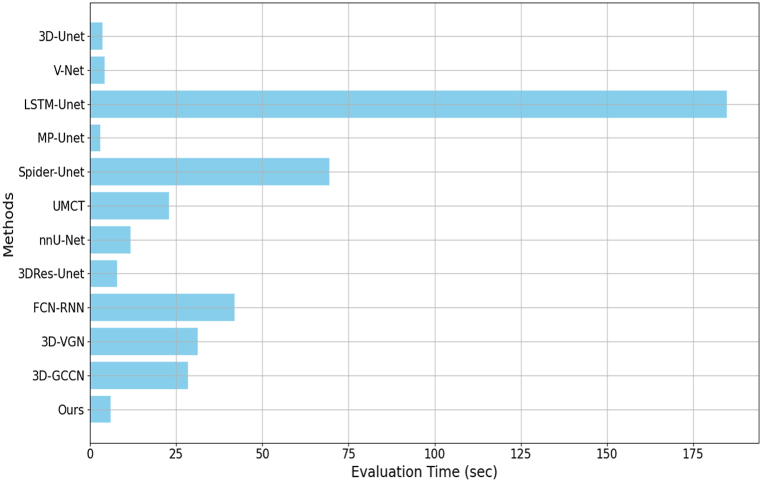
Experimental results comparison on evaluation time.

To further evaluate the differences in efficiency of different segmentation methods, the evaluation time of various segmentation methods is carefully analyzed and described in a histogram format. In the conducted evaluation, various segmentation methods were compared as measured by the evaluation time in seconds. As illustrated in Fig. 5, among the methods evaluated, LSTM-Unet required a considerably longer evaluation time of 184.79 s, reflecting its intensive computational demand. In stark contrast, MP-Unet demonstrated exceptional efficiency, clocking in at just 2.98 s, the most expedient among the methods assessed. Our approach, while not achieving the lowest evaluation time, recorded a time of 5.97 s, surpassing the times of 3D-Unet, V-Net, and MP-Unet. Despite the relatively extended evaluation time of our method, it excelled significantly in terms of the Dice Coefficient, a paramount metric for assessing segmentation accuracy. This marked superiority in accuracy delineates the efficacy of our approach, particularly in contexts where precision is of utmost importance. Consequently, when juxtaposing both the evaluation time and the Dice Coefficient, our method emerges as the most advantageous among all the methods compared. This amalgamation of efficiency and unparalleled accuracy accentuates the potential of our approach for practical applications in medical image segmentation, where both accuracy and computational efficiency are essential for clinical applicability.

## 6 Conclusion

A liver vessel segmentation algorithm based on an improved V-Net is proposed to automatically segment liver vessels with a small number of training samples and incomplete annotations. The methods such as mirroring and flipping are adopted in the process of preprocessing to increase the amount of data. The encoder of the network model exploits the dilated convolution to improve the receptive field while reducing downsampling. By using pyramidal convolution blocks, the localization ability of the network is further improved; multi-resolution deep supervision is introduced into the improved network, and liver vessel segmentation is regarded as multi-task learning, that is, liver vessels are divided into multi-resolution feature maps, so that each path There are specific training objectives under deep supervision and do not interfere with each other, thus increasing segmentation robustness. Finally, different multi-resolution feature maps are fused to predict the overall segmentation results. Post-processing operations are used in the prediction stage to further increase the prediction accuracy, and finally achieve precise segmentation of liver vessels. Through the above improvements, the precise segmentation of liver vessels can be realized.

The efficacy of the proposed method in this paper has been demonstrated on a publicly available dataset of liver vessels. Compared with mainstream network models, our proposed network model outperforms other network models. The proposed network model can accurately and quickly segment the hepatic blood vessels with complex structures from CT images. And the final resulting segmentation is more clinically relevant. While recognizing the limitations of our current approach, including dependence on the quality and diversity of training data, the need for broader validation of its generalizability, and performance improvements, our future research will pivot to three key areas. Firstly, given the high cost of annotating liver tumor images, we aim to investigate the use of synthetic tumors [45] for training classifier models. This approach holds promise for drastically reducing deployment costs and enhancing efficiency, making it a compelling option for clinical application. Secondly, the emphasis will be on the critical role of explainable AI (XAI) models [46] in medical fields, particularly in medical image processing, due to the significant impact of diagnostic outcomes on patient health and safety. Thirdly, we plan to enhance model performance by integrating traditional and neural methods, with a special focus on exploring transformer-based models [47] for the automatic segmentation of liver tumor images.

## Data availability statement

All data used to support the findings of the study is available upon reasonable request.

## Funding statement

This work was supported by the Research Project of Shenzhen Polytechnic University (Granted No.s 6023310034K, 6023310026K, 6021320035K), the Guangdong Provincial Department of Education Characteristic Innovation Project (Granted No. 2022KTSCX307), and the Natural Science Foundation of Guangdong Province (Granted No. 2022A1515010820).

## CRediT authorship contribution statement

**Zhendong Song:** Conceptualization. **Huiming Wu:** Writing – original draft, Methodology. **Wei Chen:** Data curation. **Adam Slowik:** Software.

## Declaration of competing interest

The authors declare that they have no known competing financial interests or personal relationships that could have appeared to influence the work reported in this paper.

## References

[1] Al-Kadi O.S. (2008, July). 4th IET International Conference on Advances in Medical, Signal and Information Processing-MEDSIP 2008.

[2] Zhang X. (2009, July). Boosting twin support vector machine approach for MCs detection. 2009 Asia-Pacific Conference on Information Processing, IEEE.

[3] Havaei M., Davy A., Warde-Farley D., Biard A., Courville A., Bengio Y., Larochelle H. (2017). Brain tumor segmentation with deep neural networks. Med. Image Anal..

[4] Zhou S.K., Greenspan H., Davatzikos C., Duncan J.S., Van Ginneken B., Madabhushi A., Summers R.M. (2021). A review of deep learning in medical imaging: imaging traits, technology trends, case studies with progress highlights, and future promises. Proc. IEEE.

[5] Sarvamangala D.R., Kulkarni R.V. (2022). Convolutional neural networks in medical image understanding: a survey. Evol. Intell..

[6] Kitrungrotsakul T., Han X.H., Iwamoto Y., Foruzan A.H., Lin L., Chen Y.W. (2017, March). Robust hepatic vessel segmentation using multi deep convolution network. Medical Imaging 2017: Biomedical Applications in Molecular, Structural, and Functional Imaging, SPIE.

[7] Ronneberger O., Fischer P., Brox T. (2015). Medical Image Computing and Computer-Assisted Intervention–MICCAI 2015: 18th International Conference, Munich, Germany, October 5-9, 2015, Proceedings, Part III 18.

[8] Çiçek Ö., Abdulkadir A., Lienkamp S.S., Brox T., Ronneberger O. (2016). Medical Image Computing and Computer-Assisted Intervention–MICCAI 2016: 19th International Conference, Athens, Greece, October 17-21, 2016, Proceedings, Part II 19.

[9] Milletari F., Navab N., Ahmadi S.A. (2016, October). 2016 Fourth International Conference on 3D Vision (3DV).

[10] Yu W., Fang B., Liu Y., Gao M., Zheng S., Wang Y. (2019, September). 2019 IEEE International Conference on Image Processing (ICIP).

[11] He K., Zhang X., Ren S., Sun J. (2016). Proceedings of the IEEE Conference on Computer Vision and Pattern Recognition.

[12] Salehi S.S.M., Erdogmus D., Gholipour A. (2017). Machine Learning in Medical Imaging: 8th International Workshop, MLMI 2017, Held in Conjunction with MICCAI 2017, Quebec City, QC, Canada, September 10, 2017, Proceedings 8.

[13] Thomaz R.L., Carneiro P.C., Bonin J.E., Macedo T.A.A., Patrocinio A.C., Soares A.B. (2016). XXV Congresso Brasileiro de Engenharia Biomédica.

[14] Tan S., Li L., Choi W., Kang M.K., D D'Souza W., Lu W. (2017). Adaptive region-growing with maximum curvature strategy for tumor segmentation in 18F-FDG PET. Phys. Med. Biol..

[15] Arica S., Avşar T.S., Erbay G. (2018, November). 2018 Medical Technologies National Congress (TIPTEKNO).

[16] Zeng Y.Z., Liao S.H., Tang P., Zhao Y.Q., Liao M., Chen Y., Liang Y.X. (2018). Automatic liver vessel segmentation using 3D region growing and hybrid active contour model. Comput. Biol. Med..

[17] Chartrand G., Cresson T., Chav R., Gotra A., Tang A., De Guise J.A. (2017). Liver segmentation on CT and MR using Laplacian mesh optimization. IEEE Trans. Biomed. Eng..

[18] Osher S., Sethian J.A. (1988). Fronts propagating with curvature-dependent speed: algorithms based on Hamilton-Jacobi formulations. J. Comput. Phys..

[19] Zheng Z., Zhang X., Xu H., Liang W., Zheng S., Shi Y. (2018). A unified level set framework combining hybrid algorithms for liver and liver tumor segmentation in CT images. BioMed Res. Int..

[20] Li C., Wang X., Eberl S., Fulham M., Yin Y., Chen J., Feng D.D. (2013). A likelihood and local constraint level set model for liver tumor segmentation from CT volumes. IEEE Trans. Biomed. Eng..

[21] Li Y., Zhao Y.Q., Zhang F., Liao M., Yu L.L., Chen B.F., Wang Y.J. (2020). Liver segmentation from abdominal CT volumes based on level set and sparse shape composition. Comput. Methods Progr. Biomed..

[22] Vivanti R., Ephrat A., Joskowicz L., Lev-Cohain N., Karaaslan O.A., Sosna J. (2015). Patch-Based Techniques in Medical Imaging: First International Workshop, Patch-MI 2015, Held in Conjunction with MICCAI 2015, Munich, Germany, October 9, 2015, Revised Selected Papers 1.

[23] Wang X.P., Zhang W., Cui Y. (2015). Tumor segmentation in lung CT images based on support vector machine and improved level set. Optoelectron. Lett..

[24] Zhang J., Xie Y., Wu Q., Xia Y. (2019). Medical image classification using synergic deep learning. Med. Image Anal..

[25] Litjens G., Kooi T., Bejnordi B.E., Setio A.A.A., Ciompi F., Ghafoorian M., Sánchez C.I. (2017). A survey on deep learning in medical image analysis. Med. Image Anal..

[26] Romero F.P., Diler A., Bisson-Gregoire G., Turcotte S., Lapointe R., Vandenbroucke-Menu F., Kadoury S. (2019, April). 2019 IEEE 16th International Symposium on Biomedical Imaging (ISBI 2019).

[27] Ghoneim A., Muhammad G., Hossain M.S. (2020). Cervical cancer classification using convolutional neural networks and extreme learning machines. Future Generat. Comput. Syst..

[28] Liang L., Sheng X., Lan Z., Yang G., Chen X. (2019). U-shaped retinal vessel segmentation algorithm based on adaptive scale information. Acta Opt. Sin..

[29] Li R., Li M., Li J., Zhou Y., Connection sensitive attention U-NET for accurate retinal vessel segmentation, arXiv preprint arXiv:1903.05558 (2019), pp. 1-10.

[30] Hu J., Shen L., Sun G. (2018). Proceedings of the IEEE Conference on Computer Vision and Pattern Recognition.

[31] Pereira S., Pinto A., Amorim J., Ribeiro A., Alves V., Silva C.A. (2019). Adaptive feature recombination and recalibration for semantic segmentation with fully convolutional networks. IEEE Trans. Med. Imag..

[32] Hu H.X., Mao W.J., Lin Z.Z., Hu Q., Zhang Y. (2021). Multimodal brain tumor segmentation based on an intelligent UNET-LSTM algorithm in smart hospitals. ACM Trans. Internet Technol..

[33] Tetteh G., Efremov V., Forkert N.D., Schneider M., Kirschke J., Weber B., Menze B.H. (2020). Deepvesselnet: vessel segmentation, centerline prediction, and bifurcation detection in 3-D angiographic volumes. Front. Neurosci..

[34] Peng C., Zhang X., Yu G., Luo G., Sun J. (2017). Proceedings of the IEEE Conference on Computer Vision and Pattern Recognition.

[35] Dou Q., Yu L., Chen H., Jin Y., Yang X., Qin J., Heng P.A. (2017). 3D deeply supervised network for automated segmentation of volumetric medical images. Med. Image Anal..

[36] Simpson A.L., Antonelli M., Bakas S., Bilello M., Farahani K., Van Ginneken B., … Cardoso M.J., A large annotated medical image dataset for the development and evaluation of segmentation algorithms, arXiv preprint arXiv:1902.09063 (2019), pp. 1-15.

[37] Lin T.Y., Goyal P., Girshick R., He K., Dollár P. (2017). Proceedings of the IEEE International Conference on Computer Vision.

[38] Le C.T., Wang D., Villanueva R., Liu Z., Hammer D.X., Tao Y., Saeedi O.J. (2021). Novel application of long short-term memory network for 3D to 2D retinal vessel segmentation in adaptive optics—optical coherence tomography volumes. Appl. Sci..

[39] Perslev M., Dam E.B., Pai A., Igel C. (2019). Medical Image Computing and Computer Assisted Intervention–MICCAI 2019: 22nd International Conference, Shenzhen, China, October 13–17, 2019, Proceedings, Part II 22.

[40] Lee K., Sunwoo L., Kim T., Lee K.J. (2021). Spider U-Net: incorporating inter-slice connectivity using LSTM for 3D blood vessel segmentation. Appl. Sci..

[41] Xia Y., Liu F., Yang D., Cai J., Yu L., Zhu Z., Roth H. (2020). Proceedings of the IEEE/CVF Winter Conference on Applications of Computer Vision.

[42] Isensee F., Jaeger P.F., Kohl S.A., Petersen J., Maier-Hein K.H. (2021). nnU-Net: a self-configuring method for deep learning-based biomedical image segmentation. Nat. Methods.

[43] Shin S.Y., Lee S., Yun I.D., Lee K.M. (2019). Deep vessel segmentation by learning graphical connectivity. Med. Image Anal..

[44] Li R., Huang Y.J., Chen H., Liu X., Yu Y., Qian D., Wang L. (2021). 3d graph-connectivity constrained network for hepatic vessel segmentation. IEEE J. Biomed. Health Inform.

[45] Hu Q., Chen Y., Xiao J., Sun S., Chen J., Yuille A.L., Zhou Z. (2023). Proceedings of the IEEE/CVF Conference on Computer Vision and Pattern Recognition.

[46] La Gatta V., Moscato V., Postiglione M., Sperlì G. (2021). PASTLE: pivot-aided space transformation for local explanations. Pattern Recognit. Lett..

[47] Chakraborty T., La Gatta V., Moscato V., Sperlì G. (2023). Information retrieval algorithms and neural ranking models to detect previously fact-checked information. Neurocomputing.

